# Results from Reindl *et al*. (2025) support rather than challenge the sequence hypothesis

**DOI:** 10.1098/rsos.251627

**Published:** 2025-11-12

**Authors:** Johan Lind, Andreas Wartel, Anna Jon-And, Axel Ekström, Vera Vinken, Magnus Enquist

**Affiliations:** ^1^ Centre for Cultural Evolution, Department of Psychology, Stockholm University, Stockholm, Sweden; ^2^ Division of Biology, Linköping University, Linköping, Sweden; ^3^ Department of Bird Ringing and Palynology, Swedish Museum of Natural History, Stockholm, Sweden; ^4^ Department of Romance Studies and Classics, Stockholm University, Stockholm, Sweden; ^5^ Speech, Music & Hearing, KTH Royal Institute of Technology, Stockholm, Sweden; ^6^ Biosciences Institute, Newcastle University, Newcastle upon Tyne, UK; ^7^ Department of Zoology, Stockholm University, Stockholm, Sweden

**Keywords:** sequence discrimination, sequence hypothesis, human evolutionary transition, animal intelligence, sequence memory

Understanding mental differences between humans and other animals may inform us about the preconditions that enabled human culture on a grand scale. The human capacity to faithfully represent stimulus sequences has been argued to represent one such trait [1–3]. The hypothesis (hereafter the sequence hypothesis) holds that an improved capacity to represent sequential information allowed humans to process information in novel ways (see hypothesis 5 and chapter 5 in [1]). We welcome the recent publication by Reindl and colleagues, which challenged the sequence hypothesis by subjecting capuchins, squirrel monkeys, chimpanzees and humans to a novel and creative sequence discrimination task with impressively large sample sizes [4]. Here, we reiterate their main results, outline why their study did not test the sequence hypothesis and conclude what consequences their study has for this hypothesis.

Reindl and colleagues argue that their study indicates that non-human animals’ sequence discrimination abilities have been underestimated in results that laid the foundation for the sequence hypothesis. Overall, the literature on sequence discriminations has shown that all tested non-human animals, for example, pigeons, dogs, songbirds, parrots and great apes—(i) struggle in learning to discriminate between sequences of stimuli [2,3], and (ii) their performance in sequence discrimination tasks varies systematically with details of stimulus presentations and is well accounted for by a trace memory model, a model based on well-established findings from studies of animal memory [2]. Importantly, this model can explain seemingly paradoxical findings of non-human animals both failing and succeeding in remembering stimulus sequences [5].

Reindl and colleagues argue that one reason for non-human animals’ poor performance on stimulus sequence discrimination tasks is that stimuli and their presentations have been arbitrary (see their text for details). We welcome approaches striving to improve animals’ performance in these tasks, so we can better evaluate the sequence hypothesis. We also agree with the authors that it would be problematic if animals’ sequence memory abilities are systematically underestimated. However, it is well known that discrimination studies can be made easier or more difficult in a number of ways [6]. Previous studies have overall used salient stimuli that animals perceive with ease, coupled with food rewards to enable learning about stimuli. In addition, we are not aware of any evidence suggesting animals are inferior to humans in discriminating between single stimuli, even arbitrary ones [1]. To improve sequence discrimination methodology, Reindl *et al.* attempted to create experimental conditions where stimulus sequences were explicitly linked to functional outcomes, and specific demonstrations by a human experimenter should make test subjects more attentive to the task (see their study for details). However, Reindl *et al.* only speculate, not show, that previous studies have underestimated animals’ performance and that their methodology presents an improvement.

In short, Reindl *et al*.’s study involved two training phases and one test phase. During test trials, a human experimenter placed two items, one after the other, into an opaque tube: a paper towel ball (stimulus A) or a high-value reward (stimulus B). After this presentation, subjects could either point to a small external reward or towards the tube. Pointing to the tube was considered correct only in AB trials, where the paper towel was placed first and the high-value reward followed, because this choice made the high-value reward available. In the other sequences, AA, BA or BB, pointing to the small external reward, was counted as correct. Tests were carried out under two conditions. In the ‘causal condition’, if the subject pointed to the tube, the experimenter turned it upside down, and after an AB trial, this released the high-value reward. In the ‘no-causal condition’, the experimenter used a separate box to make the high-value reward available, instead of manipulating the tube.

Squirrel monkeys (*n* = 23) and capuchins (*n* = 24) did not enter the test phase because they did not pass the second training phase. Of 13 chimpanzees, three completed all test sessions of 300 trials, but no chimpanzee learned the task to criterion within those trials. After prolonged testing, one individual reached criterion after 324 trials. Although human subjects did not receive identical treatments, they reached criterion substantially faster than the single chimpanzee who eventually did. In conclusion, these sequential tasks posed considerable difficulty for monkeys and apes, lending further support to the sequence hypothesis.

Reindl *et al.* did not consider the obvious alternative, that performance in monkeys and apes may be accounted for by the above-mentioned trace memory model. This model of sequence representation in non-human animals not only predicts whether sequences can be told apart or not, but also how it is achieved. It is based on the idea of memory traces, and it specifies quantitatively when sequence discriminations are easier to learn and when they are harder [2]. Trace memory can be powerful, recruit relevant information, and cut learning costs [7]. In contrast, humans learn to encode and recall high-fidelity representations of stimulus sequences in very few trials [3]. It would be interesting to explore what Reindl *et al*.’s results imply for the trace memory model, because comparisons of different sequence discriminations (e.g. AB versus A, AB versus BA and AB versus B) allow more powerful tests than studying the number of trials to an arbitrary criterion. A trace memory would have increasing difficulties with these discriminations in the given order, whereas they are more or less of equal difficulty for a sequence memory. Unfortunately, this is not discussed in their study.

Seen in the light of the studies discussed above, the results of Reindl and colleagues’ study do not challenge the sequence hypothesis. Rather, it corroborates previous findings that non-human animals indeed struggle to represent and recall sequential information. Finally, despite the points above, we hope this impressive study will encourage researchers to continue developing innovative approaches to testing this hypothesis.

## Data Availability

This article has no additional data.

## References

[1] Enquist M, Ghirlanda S, Lind J. 2023 The human evolutionary transition: from animal intelligence to culture. Princeton, NJ: Princeton University Press.

[2] Ghirlanda S, Lind J, Enquist M. 2017 Memory for stimulus sequences: a divide between humans and other animals? R. Soc. Open Sci. **4**, 161011. (10.1098/rsos.161011) 28680660 PMC5493902

[3] Lind J, Vinken V, Jonsson M, Ghirlanda S, Enquist M. 2023 A test of memory for stimulus sequences in great apes. PLoS One **18**, 1–16. e0290546. (10.1371/journal.pone.0290546) PMC10482264 37672549

[4] Reindl E, Seed AM, Barton RA, Francis-Costa T, Kendal RL. 2025 Humans may not have a uniquely enhanced sequence memory: sequence discrimination is facilitated by causal-logical framing in humans and chimpanzees. R. Soc. Open Sci. **12**, 250236. (10.1098/rsos.250236) 40727398 PMC12303101

[5] Lind J, Jon-And A. 2025 A sequence bottleneck for animal intelligence and language? Trends Cogn. Sci. **29**, 242–254. (10.1016/j.tics.2024.10.009) 39516147

[6] Dickinson A. 1994 Instrumental conditioning. In Animal learning and cognition (ed. N Mackintosh), pp. 45–79. San Diego, CA: Elsevier. (10.1016/B978-0-08-057169-0.50009-7)

[7] Jon-And A, Jonsson M, Lind J, Ghirlanda S, Enquist M. 2023 Sequence representation as an early step in the evolution of language. PLoS Comput. Biol. **19**, 1–19. e1011702. (10.1371/journal.pcbi.1011702) PMC10752568 38091352

